# Evidence for the Continued Use of Medieval Medical Prescriptions in the Sixteenth Century: A Fifteenth-Century Remedy Book and its Later Owner

**DOI:** 10.1017/mdh.2016.1

**Published:** 2016-04

**Authors:** Margaret Connolly

**Affiliations:** School of English, University of St Andrews, St Andrews, Fife KY16 9AL, UK

**Keywords:** Remedy book, Medieval recipes, Bodley Rawlinson c. 299, Thomas Roberts, Plague, Charms

## Abstract

This article examines a fifteenth-century remedy book, Oxford, Bodleian Library, Rawlinson c. 299, and describes its collection of 314 medieval medical prescriptions. The recipes are organised broadly from head to toe, and often several remedies are offered for the same complaint. Some individual recipes are transcribed with modern English translations. The few non-recipe texts are also noted. The difference between a remedy book and a leechbook is explained, and this manuscript is situated in relation to other known examples of late medieval medical anthologies. The particular feature that distinguishes Oxford, Bodleian Library, Rawlinson c. 299 from other similar volumes is the evidence that it continued to be used during the sixteenth century. This usage was of two kinds. Firstly, the London lawyer who owned it not only inscribed his name but annotated the original recipe collection in various ways, providing finding-aids that made it much more user-friendly. Secondly, he, and other members of his family, added another forty-three recipes to the original collection (some examples of these are also transcribed). These two layers of engagement with the manuscript are interrogated in detail in order to reveal what ailments may have troubled this family most, and to judge how much faith they placed in the old remedies contained in this old book. It is argued that the knowledge preserved in medieval books enjoyed a longevity that extended beyond the period of the manuscript book, and that manuscripts were read and valued long after the advent of printing.

Modern attitudes to knowledge in general and health care in particular place the highest value on new advances, discoveries, and techniques, and tend to regard older methods as old-fashioned and liable to be outdated. We look for currency in medical practice and seek reassurance that treatments are the most modern; something that is ‘old’ in medical terms is liable to be little valued. In terms of sources of written information, we seek out the most recently published works or the newest editions of old ones; second and subsequent editions render earlier versions mostly obsolete, and ‘new’ is synonymous with ‘better, improved’. This prevailing attitude is especially true in scientific and medical subjects, but even in the arts and humanities a high value is allocated to new ideas.

Gaining access to the new has never been easier as online information increasingly displaces the printed work as the place of first resort. As a consequence the value of ordinary old books (that is, books that are not old and rare enough to be objects of interest to antiquarians and specialist collectors) has probably never been lower. This is a recent development, and one that is easily overstated, since even now the internet has not completely displaced written sources of knowledge; books and online resources continue to coexist, and, in the field of practical information in particular, the medical encyclopedia, the DIY manual, and all manner of cookery books are still useful items on the family bookshelf.

In England in the sixteenth century a similarly visible dichotomy between old and new means of accessing information was increasingly emerging as printing, introduced to England in 1476, became more widespread. The intrinsic nature of books changed: previously they had been rare acquisitions that were labour-intensive, slow, and expensive to produce; now they became more available because of the comparative ease of printing. Early print runs were very small, to minimise the printer’s outlay, but a popular work could be quickly and frequently reprinted. The inclusion of information about the date and place of production allowed readers a new awareness of details of publication that had been lacking from manuscript books, and, as printed copies became more common, manuscripts must have looked increasingly old-fashioned. Yet, despite this modernisation, there is clear evidence that the knowledge contained in old books was still highly valued. In general terms the sheer survival of significant numbers of medieval manuscripts attests to this, especially humble, non-decorated, non-illuminated volumes. More particularly, the evidence added to the leaves of medieval manuscripts by sixteenth-century readers demonstrates very clearly how these later owners and readers regarded and used their old medieval books. This is true of all types of volumes including medical and scientific manuscripts. Analysis of readers’ annotations has become a standard practice amongst historians of the book, and over the last few decades there has been a great deal of interest devoted to marginalia. The focus of this attention, especially amongst Renaissance scholars, has been directed towards the study of ‘adversaria’ (handwritten notes in printed books), resulting in the publication of both individual case studies and overviews.^1^ Many of these relate to the reading of significant writers of the period or to annotations made to copies of literary writings.^2^ Less attention has been paid to contemporary readers of a less elevated status, or to early modern annotations made not in the pages of printed books but in the leaves of medieval manuscripts. This article will offer a case study that fills this gap by focusing on a manuscript collection of fifteenth-century medical recipes and its sixteenth-century owner; that this owner was a London lawyer and a gentleman might help to redress the notion that the collecting of medical recipes was a distinctly feminine undertaking in the early modern period.^3^

## 1 The Fifteenth-Century Remedy Collection

The medieval manuscript in question is Oxford, Bodleian Library, MS Rawlinson c. 299 (hereafter BodL Rawlinson c. 299). This small parchment volume measures 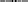 and consists of 53 folios. The original core of the manuscript (the section now numbered ff. 4r–41v) comprises five gatherings of eight leaves, though two leaves are now missing from the second gathering. This original section of the manuscript was written in the first half of the fifteenth century by a small, neat hand; the parchment has been properly prepared with pricking and ruling, and the layout of the page is professional, with a clearly defined text space, catchwords, and initial capitals rubricated in red and blue ink. Three other parchment leaves at the beginning were originally blank except for the second which has a partial copy of a uroscopy text written by a different fifteenth-century hand.^4^ The reverse of this second leaf is still blank, but the other leaves at the front of the manuscript have been filled with later additions. At the back of the book other leaves were added in the sixteenth century to augment the original volume. The quality and size of these parchment leaves vary; some of these added leaves remain blank, whilst others have been written on by a variety of sixteenth-century hands.

The text copied in the original fifteenth-century portion of the manuscript is a collection of medical recipes and treatments for many ailments. The recipes are broadly organised *de capite ad pedem* (from head to foot), beginning with treatments for headache and other afflictions that might affect the head (sunstroke, migraine, nits), moving on to the ears, eyes, and teeth (earache, deafness, tinnitus, poor eyesight, cataracts, toothache). The collection proceeds generally downwards, working through the most common problems that might affect both sexes, including fevers, kidney stones, infertility, irregular or painful menstruation, and childbirth, though the top-to-toe framework is not rigorously upheld: for example, remedies for aching in the legs and feet (f. 15v) precede afflictions of the stomach. Altogether there are 314 individual recipes in this original part of the volume. The recipes typically outline the medical problem in their opening words, advise what substances should be used and how they should be prepared, and then give instructions as to application. For example, a simple recipe for headache advises:

For akyng of a manys hed: seþe rue & fenell in watyr and with see watyr wasch þe hed.

*For aching of a man’s head: boil rue and fennel in water and together with sea water use to wash the head*.^5^

Frequently more than one remedy is listed, sometimes for an ailment which is slightly different from the previous one, but often simply as an alternative treatment for the same problem. Thus the recipe for headache cited above is immediately followed by two others:

Anoþir for akyng of þe hed behynde: tak sauge and stampe it wyth þe whyte of an ey and tempre it with vinegre and mak a pastre and lay it þer to. Anoþir: tak an ey and roste it wel hard in colys and whan it is hard cleue it on two, and as hoot as he may suffryn it lay it to his hed and it xal don away þe ache.

*Another for headache at the back of the head. Take sage and pound it with egg white and mix it with vinegar to make a plaster, and lay it on the head. Another: take an egg and roast it in coals until it is hard. When it is hard, cut it in half and lay it on his head as hot as he can stand it, and it shall remove the pain*.^6^

A variety of remedies is offered, including topical applications such as washes, ointments, and plasters, and concoctions to be ingested – usually drinks, or powders to be dissolved in liquids. Some of these preparations are described in conjunction with particular ailments, but in other cases the instructions are simply given for making a certain type of prescription, such as ‘the grene oyntment’ that may be kept for forty years or the ‘oyntment of Naruale’ (both f. 24v), suggesting either that these were multipurpose cures or that their use was well known.^7^ Occasionally there is general advice as to which herbs might be most effective against a particular disease.^8^ Sometimes general dietary advice or advice on a healthy lifestyle is provided as well, in accordance with the Galenic belief that diet was a fundamental element of medicine.^9^ Thus, for example, at the end of a recipe for tinnitus (‘twynkelyng in a mans eere’) on f. 6r is a statement of things that the sufferer should avoid doing:

… and for þese sekenesse þese ben þe chef þingys þat þou shat kepe þe fro. Ete þou no garlek and kep þe wel fro þe heete of þe sunne and leue to sowpe and kep þe fro criynge and þes ben þe chef þingis þat greuyn þe sekenesse.

… *and for this sickness these are the principal things that you should keep yourself from. Eat no garlic and keep out of the heat of the sun. Do not drink, and do not cry, and these are the principal things that aggravate the sickness*.^10^

The recipes often end with reassurance about their efficacy: for example, at the end of one recipe for cataracts on f. 7r is the statement ‘for þis is a souereyn medecyne’ (*for this is a sovereign medicine*); at the end of the next recipe for the same problem is the similarly encouraging statement: ‘and þis proued a good medecyne’ (*and this is proved a good medicine*); and the Latin tag ‘Probatus’ (*Proved*) is very frequently written in abbreviated form at the end of a recipe. These comments have not been added by a user (though that may have happened at an earlier stage in the copying history of the text): instead they are written in the hand of the main scribe, and form an integral part of the medical knowledge presented.^11^

The collection is not exclusively a collection of prescriptions to cure medical problems: there are also a few texts of a diagnostic or prognostic nature. For example, three similar recipes on f. 23r–v promise to determine whether infertility is the fault of the man or the woman (the technique requires pissing on various ingredients and observing the results); and more general diagnostic advice on f. 15v claims to be able to locate cancer in flesh, sinew, or bone. A prescription for the flux on f. 14r will show after three days whether the sufferer will live or die; similarly, holding thyme under the nose of an epileptic during a fit will reveal whether the sufferer may be cured or not (f. 21r). A short text, ‘dyuers sightes of uryne’, on how to interpret the appearance of a man’s and a woman’s urine, and what this reveals about disease in other parts of the body, is given on ff. 17v–18r. Other useful knowledge is offered in a brief list of the zodiacal signs, ‘Whan þe mone is in ony signe of þe sunne’, on ff. 33v–34r. Here the names of the signs are explained in English (so Aries is a ‘wether’, *sheep*), and they are linked to both their calendar months and to the parts of the body they were supposed to govern; the climatic conditions in particular months, and their effects on man’s life are also noted.^12^ Two other texts focus on the ingredients needed for medical preparations. One discusses the best ways of gathering herbs, noting at what time of year, and also at what time of day, certain herbs should be picked, and where the best plants are to be found:

… and herbys þat ben growende in feldys ben betre þan þoo þat growyn in towne or in gardines & þo þat growyn on hillys bin þe beste …

… *and herbs that grow in fields are better than those that grow in towns or in gardens, and those that grow on hills are the best* …^13^

The other, which is the concluding item in the original sequence, outlines the properties of ingredients used in medicines, naming the strengths of different substances and defining them in humoral terms as hot, cold, dry, or wet.^14^ Although not themselves recipes, these other texts sit comfortably with the rest of the collection to which they relate quite closely: they discuss ways to identify maladies, and give more advice about ingredients and where to find them, necessary preparations, and correct application. In short, this is a coherent collection of remedies, very much the type of compendium of useful medical knowledge that members of an ordinary late medieval household might consult for advice and self-medication. Overall the contents of BodL Rawlinson c. 299 demonstrate that this was a remedy book rather than that other type of medieval medical anthology, a leechbook, which was the kind of book used by practitioners such as surgeons and barber-surgeons. In a leechbook we would expect to find texts of another kind: as well as remedies there should be diagnostic tools such as astrological tables, diagrams, texts on bloodletting, and texts that specify the auspicious days for medical treatment; BodL Rawlinson c. 299 has none of these.^15^

As a collection of medieval medical recipes BodL Rawlinson c. 299 is not particularly remarkable. Many other similar late medieval collections exist, of which the best known are probably: the *Liber de Diversis Medicinis* from Lincoln Cathedral Library MS 91; Stockholm, Royal Library, MS X.90; and London Medical Society MS 136.^16^ These are the best known examples of the genre because they have been edited and are accessible to scholars.^17^ There are many others, some located in manuscript collections that are rich in this type of material because of the interests of their founders; notable examples are the Sloane and Harley collections at the British Library, and the Ashmole and Rawlinson collections at the Bodleian Library.^18^ Glasgow University Library’s collection of manuscripts, which largely derives from the bequest of the eighteenth-century physician William Hunter, contains at least three remedy books that are not widely known.^19^ Others are only gradually coming to light through the publication of catalogues or indexes such as the *Index of Middle English Prose*.^20^ The high quantity of this type of material should not surprise us: in their own time remedy books constituted the most frequently produced type of medical text that was written in the vernacular. Their contemporary audience was a broad one: aimed at non-specialist lay readers, this type of book might equally have been consulted by a professional, whereas academic medical treatises written in Latin could hope to reach only a more specialised readership.

The feature which distinguishes the collection of medieval recipes in BodL Rawlinson c. 299 from many other similar medieval remedy books is the evidence of its consultation and use. Even more remarkably, the exact identity of the user and the period of use are also known. Many other remedy books remain more stubbornly anonymous, both in terms of their original copyists and subsequent owners and readers. Unusually in the case of the Lincoln Cathedral manuscript of the *Liber de diversis medicinis*, the scribe-compiler is identifiable as Robert Thornton, a fifteenth-century Yorkshire gentleman who assembled texts of various types into two personal anthologies, so a contemporary interest in the contents of this medical collection may be attributed to him. This is a rare example. More typically, as in the case of Glasgow University Library MS 185, a volume may show evidence of later ownership, and present other inscriptions without the accompanying detail that would allow the precise identification of the individuals mentioned.^21^

## 2 The Tudor Reader and his Annotations

The name of the scribe who copied BodL Rawlinson c. 299 is unknown, though he may have been from Norfolk.^22^ Nor is the original owner or commissioner of the volume known. What is certain, however, is that at some point towards the end of the fifteenth century, or perhaps in the early part of the sixteenth century, the volume passed into the hands of Thomas Roberts, a London lawyer and a Tudor gentleman whose property holdings were at Neasden, in the parish of Willesden in Middlesex. Thomas Roberts was born in 1470 in the reign of Edward IV. He studied law first at Clement’s Inn (one of the inns of chancery), and then after 1501 at the Inner Temple (one of the inns of court). His practice as a lawyer increased his personal wealth and brought him a range of local government commissions – as a justice of the peace, county coroner, and so on. In short, he was very typical of his age: a middle-ranking, educated professional, with the potential to be up-and-coming. By the seventeenth century the Roberts family had become the largest landowners in Willesden, but in Thomas Roberts’s time their holdings were more modest, and they were middle-ranking gentry who were prosperous rather than wealthy. Thomas Roberts became a gentleman, partly through the inheritance of family property, but also through his own augmentation of that property by marriage and purchase. He was married three times, and had twenty-four children, of whom six, three daughters and three sons, survived to adulthood; he died in 1542.^23^

BodL Rawlinson c. 299 was already an old book by the time that it came into Thomas Roberts’s hands. As there are no previous indications of ownership, it is impossible to tell whether this was a book that had been handed down within the Roberts family, or whether Thomas had newly acquired it by purchase or as a gift or bequest. As was his practice with other books that he owned, Thomas added his name frequently throughout its pages.^24^

Typically he wrote ‘Robertz’ or ‘Robertes’, often in abbreviated form in the upper margins of the text (as on ff. 12r, 21r, 24r, 25r, and 25v). Some of these inscriptions are placed at the beginnings or ends of gatherings, and may simply have been added to proclaim ownership, especially since it was common for manuscripts to exist for a considerable time as a series of unbound or lightly bound quires rather than being properly fixed within boards. More suggestively, on f. 16r Thomas wrote his name in the right margin alongside a remedy for cancer, perhaps signalling a particular interest in this recipe. He also sometimes wrote his name into small blank spaces within the text frame where the originally written text did not quite fill up the line. This occurs in five places on ff. 21r, 24r (see Figure 2), 26r, 34r, and 39r, and in each case it is not clear whether Thomas was marking the adjacent recipe texts for special interest or not: the texts in question comprise: dietary advice for epileptics (f. 21r); a prescription for ‘water de copurose’, *a metallic sulphate, vitriol* (f. 24r), recommended for a variety of complaints; a recipe for making a gummed cloth which was ‘good for brosures’, *wounds, bruises* (f. 26r); a list of the signs of the zodiac (f. 34r); and a recipe to make a drink to cure a ‘posteme’, *swelling, inflammation* (f. 39r).

Amongst other texts inscribed at the beginning of the volume, to which this discussion will return later, Thomas added one distinctly informative note, recording where a named practitioner, the proponent of a contemporary wonder drug, might be found: ‘Thomas Warde surgeon at seynt Andrewes Vndershaft can hele & help all diseases & sores with one medycine or salue’ (see Figure 3).^25^ The manuscript has one other similarly informative comment relating to its medical contents inscribed in the margin of f. 4v, alongside a recipe for lice and nits:

For lees and netys in þe heued and oþir maner of wermys in what place so euere þei been: tak quyklym and tempre it with þe ious of walwort as wasch þe place, and þei xuln sone deye probatus.

*For lice and nits in the head, and other kinds of vermin wherever they may be. Take quicklime and mix it with the juice of walwort. Wash the affected place, and the vermin will soon die. Proved*.^26^

**Figure 1:**
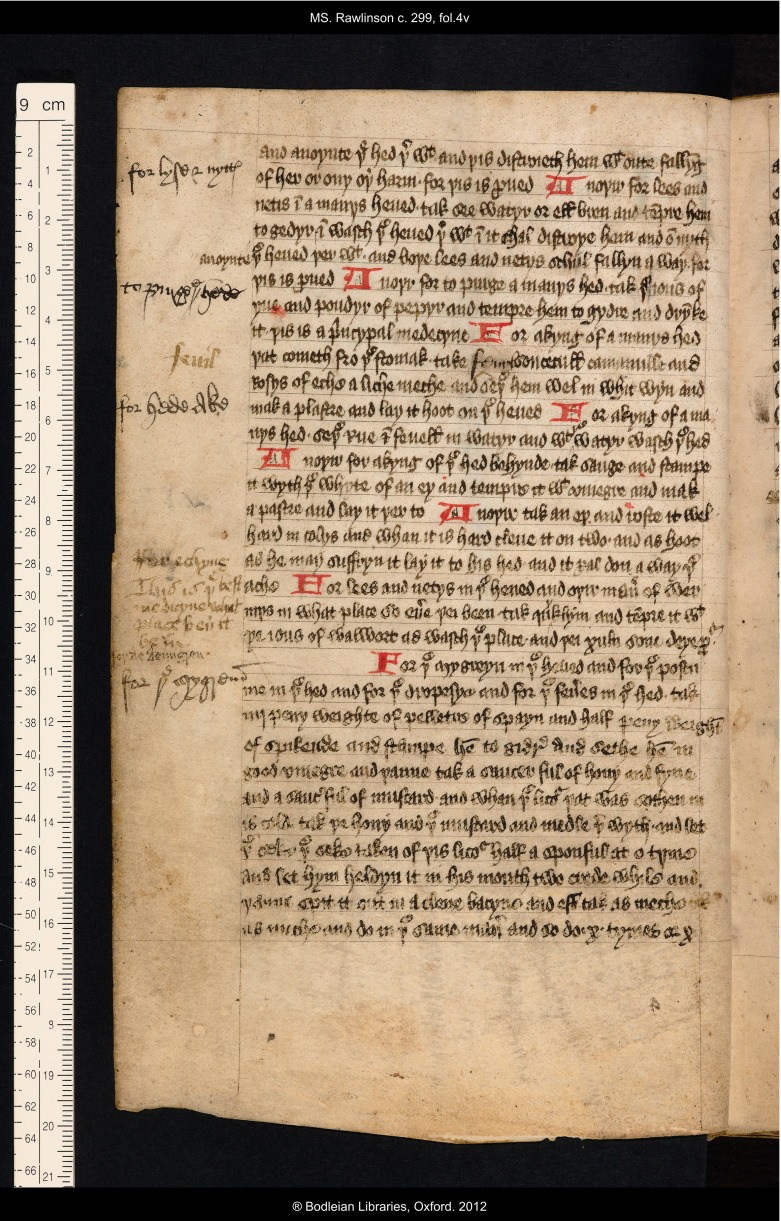
Oxford Bodleian Library, MS Rawlinson c. 299, f. 4v; recipes for headache, with marginal additions. By permission of The Bodleian Library, University of Oxford.

In the left margin is written ‘for echyng’ (*itching*) and then the comment ‘This is þe best medicyne what place ben it be in’ (see Figure 1). This comment was probably not, in fact, written by Thomas Roberts. The ink is much lighter than the black ink he habitually uses, and, although differences in ink colour often only denote different writing stints, the formation of the letters in these words is much less practised, bespeaking a more amateur annotator than Thomas with his professional legal script. Nevertheless, this is a contemporary addition, and one that clearly demonstrated that the inscriber placed value on this particular remedy. At first glance BodL Rawlinson c. 299 seems to be full of such annotations – there are additions in the margins throughout; the manuscript promises to be a rich resource for charting the later reception of medieval recipes, and it is a disappointment to find that this is the only such comment on a particular remedy.

**Figure 2:**
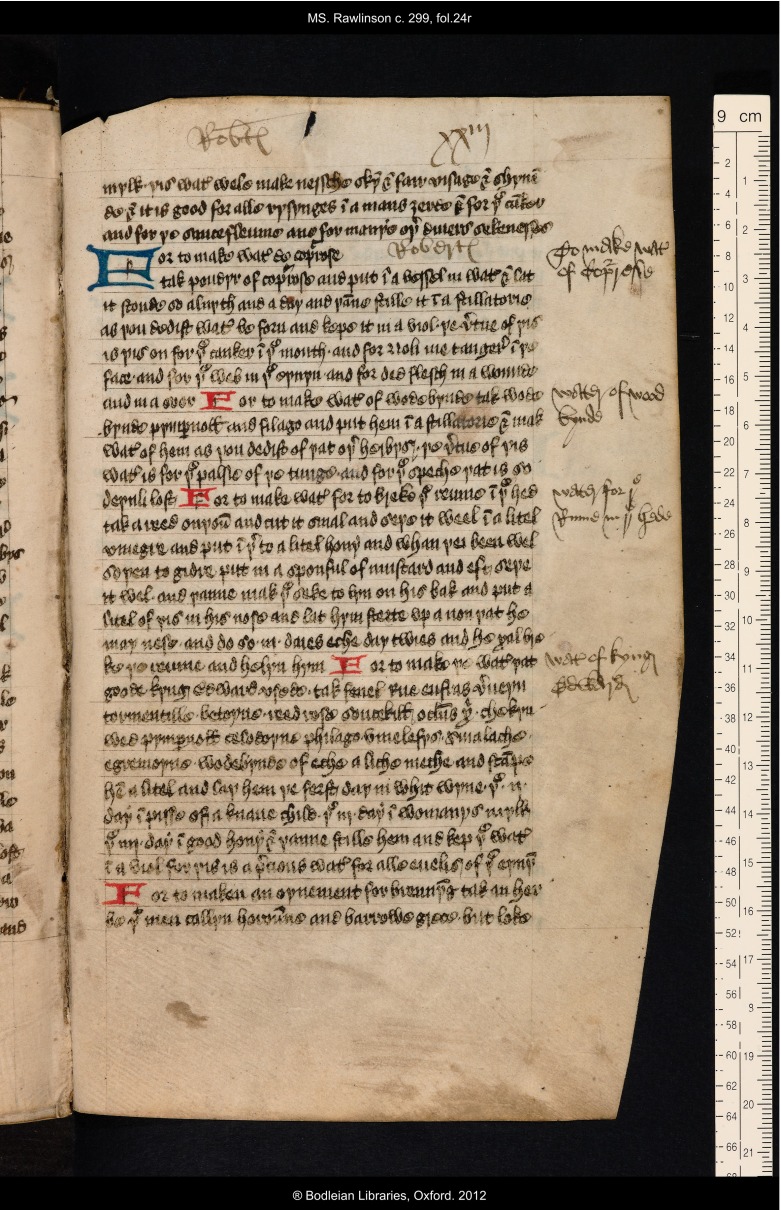
Oxford, Bodleian Library, MS Rawlinson c. 299, f. 24r, showing Thomas Roberts’s name in the upper margin and in the blank space of line 4. Note also the added headings in the right margin relating to the recipes copied in the text. By permission of The Bodleian Library, University of Oxford.

**Figure 3:**
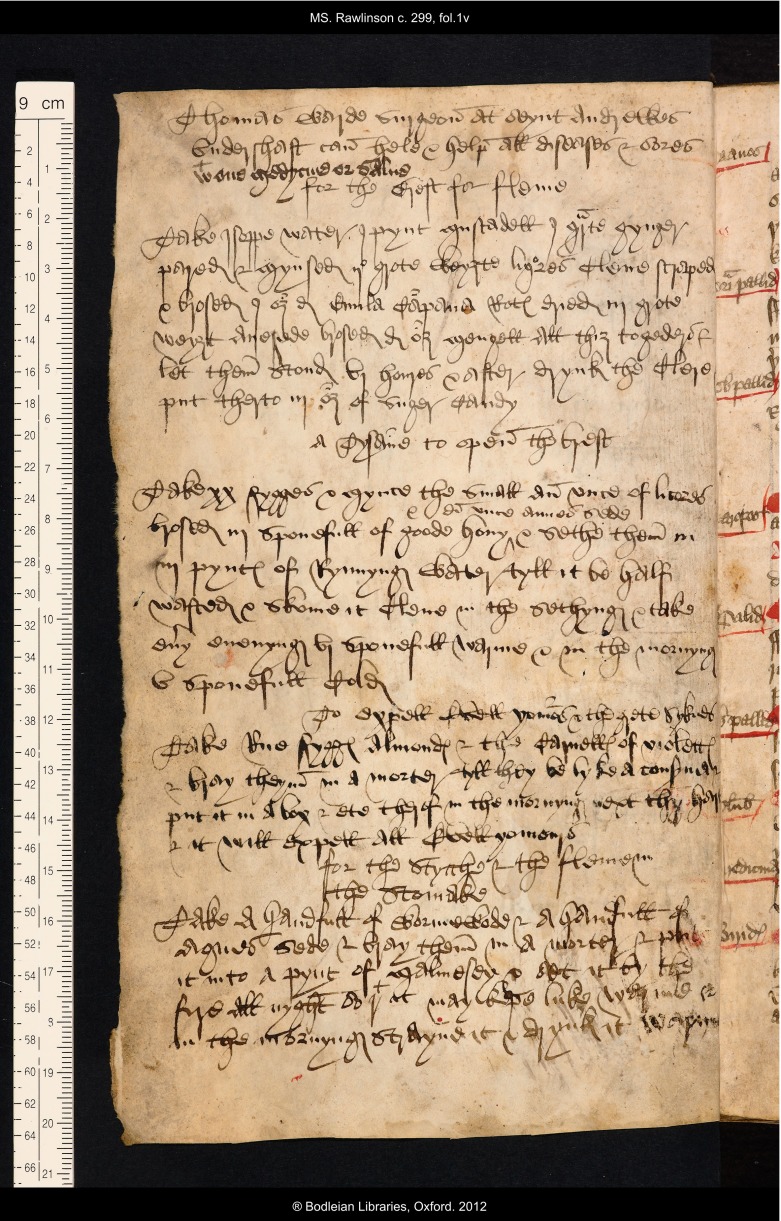
Oxford, Bodleian Library, MS Rawlinson c. 299, f. 1v; the inscription relating to Thomas Warde, surgeon, is at the top. By permission of The Bodleian Library, University of Oxford.

**Figure 4:**
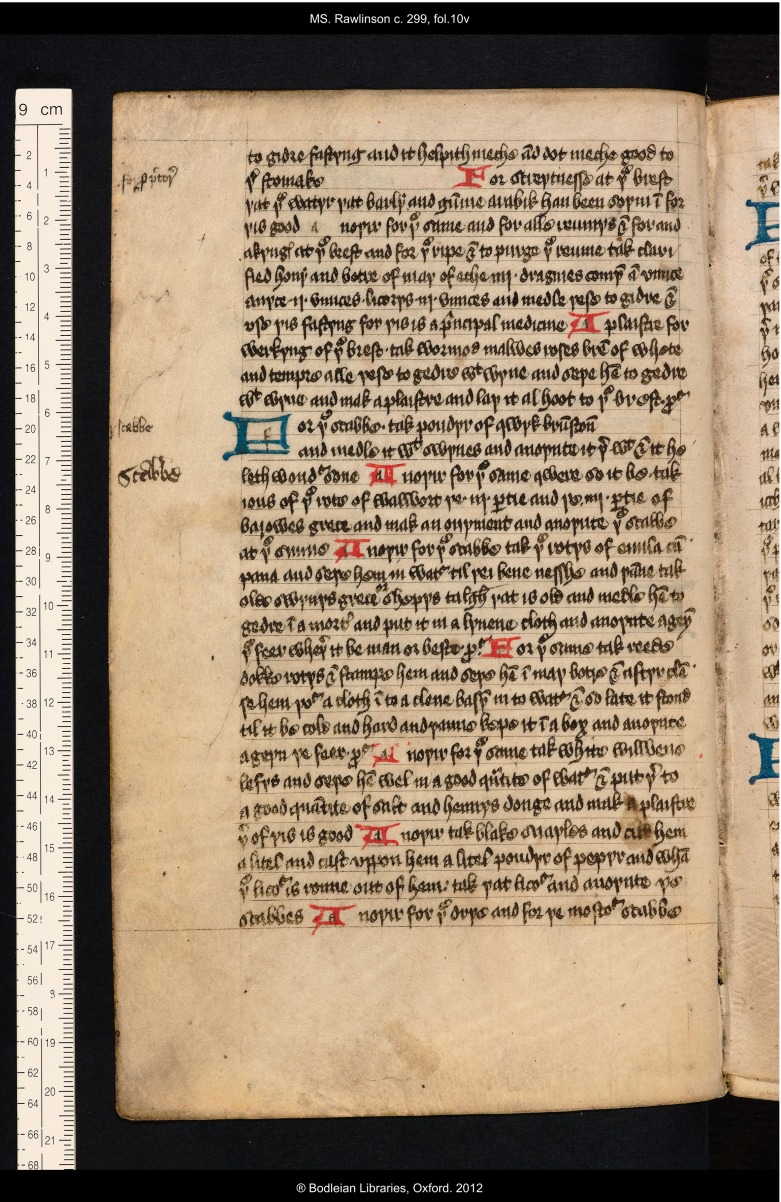
Oxford, Bodleian Library, MS Rawlinson c. 299, f. 10v; marginal annotations by two hands. By permission of The Bodleian Library, University of Oxford.

Although there are no other comments of this nature the many sixteenth-century annotations to the original remedy collection in BodL Rawlinson c. 299 reveal general evidence of sustained interest in and use of the volume in this period. The other frequent additions in the margins all prove to consist of the provision of side headings to the original recipes. Thus, for example, on f. 4v, the left margin carries the added side headings: ‘for lyse & nyttes’; ‘to purge þe hede’; ‘for hede ake’; ‘for þe mygrene’, all written by Thomas Roberts’ hand alongside the originally-written series of recipes for those complaints (see Figure 1). These side headings function as reader’s aids, enabling the searcher (Roberts himself, and other members of his household) to find the desired remedy more quickly. The margins of the volume actually present layers of such annotation, showing repeated consultation by different users, probably from successive generations of the family. On f. 4v, as well as the four side headings added by Thomas Roberts, the hand that added the comment about itching has also written the word ‘fenil’ (*fennel*) as a clarification of an ingredient listed unclearly in the ninth line of the original text.^27^ Another much smaller hand has also supplied a side heading to the remedy for migraine, writing ‘for de demigren’. Similarly layered annotations may be observed throughout the manuscript, with the side headings contributed by the much smaller hand sometimes duplicated by another; see for example the additions to the left margin of f. 10v, which consist of two annotations by the smaller hand, the second of which, ‘scabbe’ (alongside a series of remedies ‘For þe scabbe’, *skin disease*) has been emphatically repeated in a larger script (see Figure 4).

**Figure 5:**
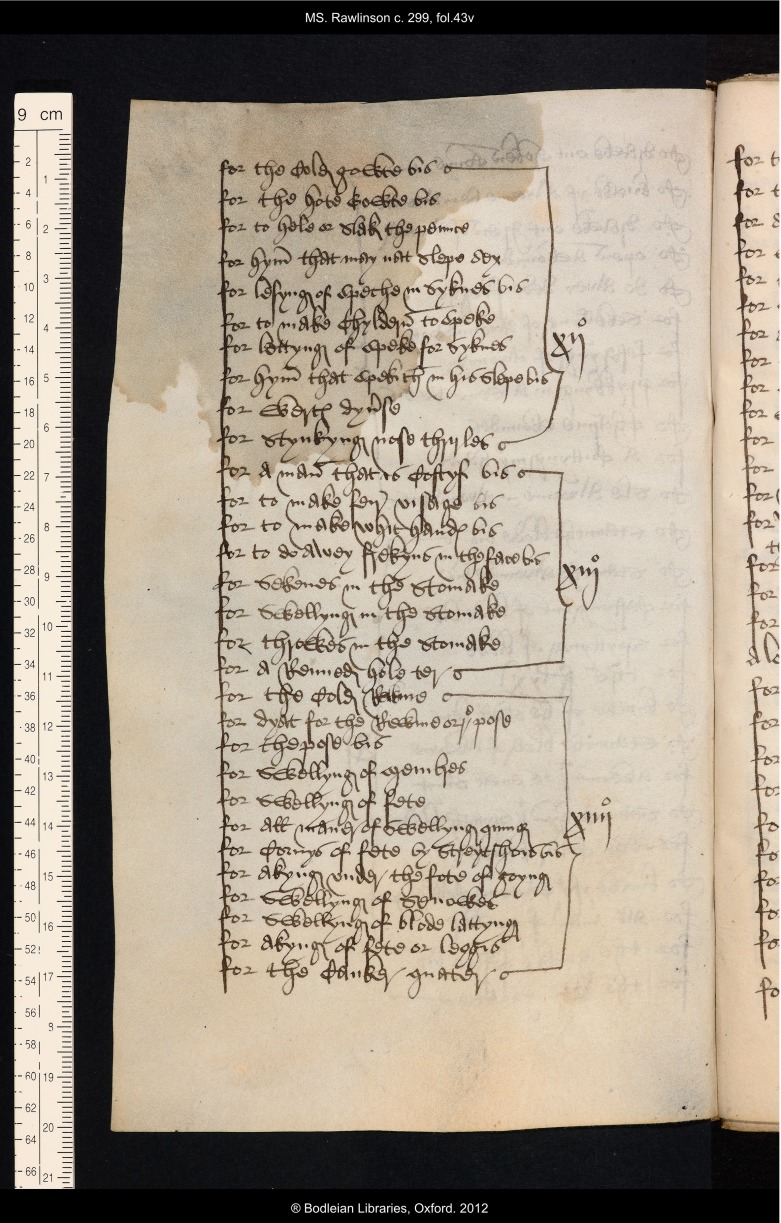
Oxford, Bodleian Library, MS Rawlinson c. 299, f. 43v; fourth page in the list of contents, showing entries relating to the pages numbered in the sixteenth century as ‘xij’, ‘xiij’ and ‘xiiij’. By permission of The Bodleian Library, University of Oxford.

The side headings added by Thomas Roberts map onto a list of the recipes that he prepared and added at the back of the original book. The list, with the heading ‘Tabula medicinarum huius libri’ (*Table of the medicines in this book*), is written on parchment leaves (ff. 42r–47r) that were added to the five original fifteenth-century gatherings. The list is arranged as a single column of entries which are bracketed together into groups linked to roman numerals; these numerals relate to the numbers that Roberts added at the top right corner of each recto leaf.^28^ Thus by consulting the list at the back of the book, a reader could quickly see what recipes were included in the whole collection, and also where to find remedies for particular ailments (see Figure 5).

When turning to the leaf in question the reader was further helped by the side headings added in the margins. The list of contents is somewhat condensed because it consists of a list of ailments rather than a complete list of remedies; not all of the different remedies for the same problem are included as Thomas used the word ‘dyuerse’ (*various, several*) to indicate that alternative recipes for the same complaint will be found. For example, whilst sixteen separate recipes appear on f. 4r–v, the relevant part of the list of contents on f. 42r has only eight entries; the first entry reads ‘for the hede ache dyuerse’, and the second is for ‘a playster for hede ache’, whereas on f. 4r the first three recipes are all for headache, as is the fourth, but that is the first to mention a plaster. Accordingly the list of contents has 287 entries even though the recipe collection itself has 314 remedies. This list of contents, and the marginal headings to which it is keyed, together constitute a user-friendly apparatus that allows efficient navigation of the recipe collection. Such strategies for the organisation and quick retrieval of information would have been instinctive to a trained lawyer, and the provision of this information management system was perhaps merely indicative of Thomas Roberts’s habitual reading practice.^29^ Nevertheless, considerable effort must have been involved in its preparation, and Thomas Roberts’s investment of his time and labour bespeaks a high regard for the recipe collection and the knowledge it contains.

Clearly this volume was a valued household manual in the first part of the sixteenth century. Its added apparatus demonstrates that regular recourse was made to the medieval recipe collection in this period, despite the fact that the texts it contained were by that time very old. It is not apparent which medieval recipes were the most often consulted, nor is it possible to tease out what particular ailments troubled Thomas Roberts and his family most severely. Sometimes signs of wear and tear betray the sections of medieval manuscripts that were of most interest to their readers: faded writing may have been damaged by repeated touching or rubbing, and dirty margins may be the result of excessive handling; medical manuscripts, in particular those that contain texts on blood-letting, are sometimes stained with what look suspiciously like bodily fluids.^30^ However, no such stains or dirt deface BodL Rawlinson c. 299; the parchment is discoloured in places, but there is nothing that cannot be explained by the normal causes of age, damp, and general handling. Only one sign of wear and tear on the manuscript is potentially significant. Two leaves, which would have been numbered xij and xiij in Thomas Roberts’s scheme, are now missing from between what are now ff. 14v–15r. That these leaves were still present in the manuscript when Thomas Roberts handled it is shown by the fact that he tabulates their recipes in his list, see Figure 5. The list therefore reveals the subject content of the missing items which were recipes for: gout; pains; sleep problems; speech problems (four recipes); warts; stinking nostrils; constipation; prescriptions to make the face fair, the hands white, and to remove freckles from the face; for sickness, swelling, and contractions in the stomach; and for discharge from the anus. Perhaps amongst this range of remedies there were some that were referred to frequently? Did heavy consultation cause this particular leaf to loosen and fall out? Its loss is a slight indication that this part of the collection might have been consulted more than others, but to gain a more reliable sense of what medical remedies were most desired by the manuscript’s sixteenth-century readers we need to investigate the recipes that they themselves added to the volume.

## 3 The Recipes Added to the Original Remedy Book

The original fifteenth-century collection of 314 recipes was augmented in the sixteenth century by the addition of a significant number of further remedies, contributed by a variety of sixteenth-century hands. These include the hand of Thomas Roberts, which can be readily identified; other hands were presumably those of other members of his family and household. These recipes, which are in varying states of completeness, were added at different times as is clear from the variety of ink shades involved. Their inscription in the volume proceeded in an accretive manner, and it is not now possible to recover the exact chronology of their addition, though variations in handwriting styles and shades of ink allow different hands and writing stints to be distinguished. In total forty-three recipes have been added to the original remedy book, nine at the front of the volume and thirty-four at the back. However, this total of forty-three may be revised downwards slightly since three of the added remedies were copied twice, two more or less verbatim, and the third copied first incompletely and then in full form. Three recipes lack specifications about the ailments to which they relate, and two are for treating horses; this veterinary material will not be considered further here.^31^ The additions therefore comprise 35 recipes, which aimed to treat the following ailments: phlegm, affecting mainly the chest but also the head and stomach (7); toothache (4); aches affecting other parts of the body (4); constipation (3); plague (3) and the sweating sickness (1); ague (3); colic (3); stone (2, one of which is also for colic); flux (2). Single recipes are inscribed for the following problems: watering of the eyes; a broken limb; a persistent sore; drunkenness. Arguably these added recipes offer the greatest insight into Tudor use of the collection. Although Thomas Roberts and his family clearly made use of the whole volume, the remedies that they themselves inscribed must have been regarded as effective and worth preserving, and must also have been ones which they had cause to use.

To a certain extent these additions make good deficiencies in the coverage of the original remedy book which offered no prescriptions to help with ague, colic, aches in the body (except in the legs and feet), broken limbs, or phlegm in the stomach. The original collection has prescriptions which promise to open or purge the breast, but none specifically to treat phlegm. This seems to have been a complaint that troubled Thomas Roberts considerably. At the beginning of the book on f. 1v he copied out four recipes for complaints of the chest and stomach (see Figure 3). The first, to cure phlegm in the chest, explains how to make a relieving drink:

Take isoppe water j pynt mustadell j quarte gynger pared & mynsed ij grote weyʒte liquores clene scraped & brosed 9 oz demi enula campania rotes dried iij grote weyʒt anesede brosed demi oz mengell all thiz togeders & let them stond vj houres & after drynk the clere & put therto iij oz of suger candy.

*Take hyssop, a pint of water, a quart of muscatel, 2 groat’s weight of ginger, pared, and minced*,  *ounces of liquorice, scraped clean and ground, a groat’s weight of dried enula campana roots, and half an ounce of ground aniseed. Mix all these together and let them stand for 6 hours. Afterwards drink the clear liquid adding 3 ounces of sugar candy*.

This recipe was obviously a favourite, because Thomas copied it again at the back of the book, on what would have been the volume’s last leaf (f. 41v) before the units of fine quality parchment on which the list of contents is copied were added. The second recipe on f. 1v is of a similar nature. Entitled ‘A tysane to open the brest’ it instructs the sufferer:

Take xx fygges & mynce the small an unce of licores brosed iij sponefull of goode hony & demi unce annes sede & sethe them in iij pyntes of rynnyng water tyll it be half wasted & skome it clene in the sethyng & take euery euenyng vj sponefull warme & in the mornyng v sponefull cold.^32^

*Take 20 figs and mince them finely, an ounce of ground liquorice, 3 spoonfuls of good honey and half an ounce of aniseed. And fast boil these in 3 pints of running water until reduced by half, and skim the liquid while it boils. And take 6 spoonfuls warm every evening and 5 spoonfuls cold in the morning*.

After a recipe for sickness, for which see below, the fourth and last recipe of this set on f. 1v is another for phlegm, this time in the stomach: ‘For the stynke & the fleme in the stomake’:

Take a handfull of wormewode & a handfull of agnes sede & bray them in a morter & put it into a pynt of malmesey & set it by the fire all nyght so þat it may kepe luke warme & in the mornyng strayne it & drynk it warme.

Take a handful of wormwood and a handful of aniseed and pound them in a mortar, and put this into a pint of malmsey (wine) and set it by the fire all night so that it stays lukewarm, and in the morning strain it and drink it warm.

Thomas added more recipes on f. 3r–v. The three on f. 3v, written in one stint as is shown from the consistency of the ink colour, are all for ‘fleme in the brest’, suggesting that Thomas may have been collecting remedies for what seems to have been a persistent problem. These recipes use similar ingredients and techniques to the remedies proposed on f. 1v:

Take a handfull of grondeselle & a pynt of Rynnyng water & a sponefull or ij of goode hony & sethe all together tyll it be wasted to half & then drynk of the liquore fastyng & it wyll make you to avoyde fleme & open the brest.

Take a handful of groundsel and a pint of running water and a spoonful or two of good honey and boil all together until the liquid be reduced by half, then drink the liquid whilst fasting and it will make you void phlegm and will open the breast.

Another for the same

Take the rote of Enulacampan fenell sede agnes a demi carwey sede liquores dry isop of eche an unce gynger half an unce & make sottell powder of all thise and put therto half a pound li of hard fyne & whit suger & ete therof at all tymes when ye haue nede & in especiall fastyng & last to bed.

Take the root of enula campana, fennel seed, half an ounce of aniseed, an ounce each of caraway seed, liquorice, and dry hyssop, half an ounce of ginger, and make a fine powder of all of these and add half a pound of fine white sugar. Eat of this at all times according to need, and especially when fasting, and last thing before bed.

Another for the same

Take half a handfull of Rue a handfull of isop ix fygges gardynn mynttes a handfull & boyll all thise in a quart of condyte water with thre sponefull of hony & skym it clene then streyn it thorugh a clen cloth into a close vessell & drynk therof half a pynt at ones blod to arme so contynue to it be done.

*Take half a handful of rue, a handful of hyssop, 9 figs, and a handful of garden mint. Boil all these in a quarter of water from the conduit with 3 spoonfuls of honey and skim the liquid, then strain it through a clean clothe into a vessel and seal. Drink half a pint at once to fortify your blood. And continue until it is finished*.

The stipulation in these recipes for the use of particular types of water – ‘running’, ‘conduit’ – demonstrates continued belief in the medicinal virtues of different waters, and a knowledge of the dangers of unclean water, as expressed in authoritative medieval texts such as Avicenna’s *The Canon of Medicine* and Bartholomaeus Anglicus’s *De proprietatibus rerum*.^33^

Another of Thomas’s additions on f. 41v is a recipe for a laxative, ‘A Goode lax’, which involves many of the same ingredients used to treat phlegm:

Take a penyworth of clene coddes a penyworth of careawey sede & a half penyworth of anys sede & sethe theym in a quart of whit wyne vnto a pynt, & then streyne them thorugh a lynen cloth & then put there a quantite of suger to make it swete, & drynk it warme fastyng, & spare mete & drynk ij or iij houres after.

Take a pennyworth of clean seed cods, a pennyworth of caraway seed, and a halfpennyworth of aniseed, and boil them in a quart of white wine reducing the liquid to a pint. Then strain them through a linen cloth and add a quantity of sugar to make it sweet. Drink it warm when fasting. Avoid meat and drink for 2–3 hours afterwards.

Amongst more recipes that he added a few leaves later, after the list of contents, is another, extremely similar ‘goode laxatyf’:

Take colyander sedes agnes sedes & clene coddes, of euery of theym a penyworth, & sethe them in ale from a quart to a pynt & clense it & make alebery therwith, thyne pot in the alebery a nutmyg & suger, & drynk therof erly & late.

*Take coriander seeds, aniseed, and clean seed cods, a pennyworth of each, and boil them in ale, reduce the liquid from a quart to a pint, and strain it, and make alebery* [heated spiced ale] *with it. Put a nutmeg and sugar in the alebery and drink this early and late.*

As with the previous recipes for phlegm, these very similar prescriptions for constipation seem to have been favourite remedies that Thomas was keen to preserve. A third added recipe for ‘a goode lax’ occurs on the very last leaf, f. 53v, and is again similar in terms of preparation and application, though with the different main ingredients of raisins and dates. The provision of these additional recipes was a useful augmentation of the existing volume which contained only one laxative, and might signal either that constipation was a common problem amongst this generation of the Roberts family, or that the condition itself was regarded more seriously at this time. Humoral theory stressed the need for an internal balance in order to maintain well-being and resist disease; when outbreaks of infection were rife the retention of potentially corrupt humoral matter within the body might be feared as a cause of debilitation, leading to a greater reliance on purgative remedies.^34^

Similarly in the original fifteenth-century remedy book there is only one recipe for use against pestilence (a drink, f. 26r), but no other remedies for plague; there are none for the sweating sickness because this was unknown in England before the outbreak of 1485.^35^ It is not at all surprising to find remedies for such problems amongst the sixteenth-century additions. The third recipe that Thomas Roberts wrote out on f. 1v was a prescription labelled: ‘To expell evell yomuros & the grete syknes’:

Take rue fygges almondes the carnelles of violettes & bray theym in a morter tyll they be lyke a conserue; put it in a box & ete therof in the mornyng next thy hart & it will expell all evell yomours.

*Take rue, figs, almonds, kernels of violets, and pound them in a mortar until they resemble a conserve. Put it in a box and eat this in the morning close to your heart and it will expel all evil humours*.

Again this must have been a valued remedy, because Thomas copied it again at the back of the book on f. 47v, where it is given the different heading ‘contra pest’ (*against the plague*).^36^ This second copy is slightly more accurate in having the reading ‘use therof in the mornyng’, rather than the contextually nonsensical ‘ete therof in the mornyng’ given on f. 1v. Other prescriptions against the ‘sweat’ and the plague are given on f. 48v. First is ‘The kynges medecyn for swetyng syknes’:

Take a sponefull of triacul a sponefull of veneger v sponefull of rynnyng water viij sponefull of juse of synkfoyll mixt theym togeder with suger & drynk it luke warme …

*Take a spoonful of theriac, a spoonful of vinegar, 5 spoonfuls of running water, 8 spoonfuls of the juice of potentilla. Mix these together with sugar and drink lukewarm* …^37^

The second is ‘For the Grete seknes pestilens’:

Take cowe mylke & sett hit ouer the fyer & when hit ys redy to seithe then put a quantitie of de lyght alome in to the milke that will make hit turne, then strayne the same & take the whaye & sett hit on the fyer & put ther in a handfull of erdes foote & lett hit seithe to hit be half consumyd, then drynk of the same whaye & yf ye haue eny risyng take the curde of the same & laye hit to the seyd rysyng & hit will gether hit & breke hit amen.^38^

Take cow’s milk and set it over the fire, and when it is ready to boil then put a quantity of light alum into the milk which will make it turn. Then strain it and take the whey and set it on the fire, and put in a handful of earth’s foot (?) and let it boil until it is reduced by half. Then drink the same whey and if you have any swelling take the curds and lay it on the swelling and it will make it rupture. Amen.

In other cases, however, the recipes that were added to the volume by its sixteenth-century users served only to duplicate aspects of the original collection where, for example, prescriptions for the stone, flux, and watering of the eyes might already be found. Indeed, the original collection has no fewer than nine remedies for drunkenness (f. 22r), so another, as offered on f. 47v, can scarcely have been needed. In several cases the added recipe not only duplicates a remedy already contained in the book, but uses the same key ingredients as the original formulation. This is the case with the remedies for flux (on f. 14r and f. 53v) which both use egg yolk, and the prescriptions to cure watering of the eyes (f. 7v and f. 53v), which both prescribe the eating of raw betony; such recipes cannot have been very different in either practice or outcome. In some instances of apparent duplication Thomas may have been recording treatments that were new or different. For example, on f. 3r he added two remedies for toothache. The fifteenth-century remedy book already contained six prescriptions to relieve toothache (ff. 8r–9r), as well as offering some advice on how to prevent the problem. Some of these recipes show similarities to the added ones in terms of preparation and method of application, but there is no equivalent amongst the fifteenth-century remedies to the ‘special gode medecyne for the totheake’ that Thomas copied on f. 3r:

Take iij croppis of rue iij sage leves ij yoyntes of Elder of one yeris shot of the middel bark that is grene, & scrape awey the first bark, & xl cornes of peper as moche salt as will into a small fut shale, & cut all theym small & bete theym all togeder, & deuyde it into iij partes & put theym into iij litell bagges of newe lynen cloth & stepe theym in aqua vite, & ley one of the bagges at the gomme ther as the tothe doth ake an houre, & so do with the other ij bagges, one after an other, & within iij houres he shal be hole & neuer haue the tothe ake more in that place wher this medecyn is leye, with Goddes grace.

Take three crops of rue, three sage leaves, 2 joints from a year-old branch of elder, of the middle bark that is green, and scrape away the first bark, and forty peppercorns, as much salt as will fill a small nut(?) shell, and cut them all small and beat them all together, and divide it into three parts, and put them into three little bags of new linen cloth and steep them in aqua vite, and put one of the bags on the gum where the tooth aches for an hour, and do the same with the other two bags, one after another, and within three hours he will be cured and will never have the toothache again in that place where this medicine was put, with God’s grace.

The method of application in the added recipe is completely different from that prescribed in any of the medieval remedies in the original collection, as are all of the ingredients with the exception of pepper.

## 4 Charms

Most of this discussion has focused on the annotations to the original fifteenth-century medical recipes in BodL Rawlinson c. 299, and on the recipes that were added to the collection by its sixteenth-century users. Investigation into who used texts and how almost always relies on the evidence of additions and other signs of use such as underlining, and even accumulated dirt and wear and tear. Yet is also worth noting some particular instances where there has been no disturbance to the original text, since occasionally there is something that may be learned even from an *absence* of activity on the part of readers.

In common with other collections of medieval recipes, BodL Rawlinson c. 299 contains a few charms. These formulations, which rely on incantation and the invocation of God, Christ, and the saints, would not now be regarded as of a medical nature at all, but their frequent preservation in medieval medical treatises and remedy books demonstrates a persistent faith in their therapeutic and healing powers.^39^ This faith was dislodged by sixteenth-century changes in religious attitudes, and as a result such texts came to be viewed as superstitious, even popish, and later readers of the Protestant persuasion frequently cancelled such texts by striking them through. None of the charms in BodL Rawlinson c. 299 has suffered this fate. The collection includes four, all of which are examples of charms that were widespread in medieval Europe. Two are for toothache: the first, in Latin, is an invocation to St Apollonia, and the second, in English, is a brief version of the ‘God was Born in Bethlehem’ charm; these both occur on f. 33v, where there is also a Latin charm to staunch blood, which is a version of the ‘Flum Jordan’ charm.^40^ The fourth and most extensive of these texts, on f. 13v, is mostly in English. Introduced as ‘For to charme woundes and þis is a principal charme & ofte proued’, it reads as follows:

Thre goode breþeren wente be þe way and hem mette Ihesus  and seyde to hem wheþer go ʒe iij goode breþeren? Lord we go to þe mount of olyuete for to gadre good herbys of saluacioun of hele. Sure to me by þe milk of seynte maris maydyn and be þe sunne and be þe mone þat ʒe xul nowt hide it in priuyte, ne ʒe xul take no mede, and go ʒe to þe mount of oliuete and tak blak wulle of a schep of two ʒer & oile de oliue and aftyr þat seyunge þus, as longeus þe knyt þe syde of oure lord ihesu  crist with a spere þer lede, and þat wounde werkede nouth rote nouth fesred nouth rankelede nouth ne bledde nouth ne made no droppyng; so þis wounde be þe vertu of þat wounde werk noth long  ne ferstre  ne rote  ne blede nouth ne make no droppyng  but be it al so hool and as clene as þe wounde þat longeus mad in þe syde of oure lord ihesu  crist whan he heng on þe cros  In nomine patris et filij et spiritus sancti amen.

Three good brothers went by the way and Jesus met them and asked them ‘Where are you going three good brothers?’ ‘Lord, we are going to the Mount of Olives to gather good herbs for the preservation of health.’ ‘Promise me by the milk of St Mary the Virgin, and by the sun, and by the moon, that you shall not keep it secret, nor shall you take any reward. And go to the Mount of Olives’. And take black wool from a two-year old sheep and olive oil. And after that saying thus: ‘As the knight Longinus laid a spear to the side of our Lord Jesus Christ, and that wound did not hurt, did not rot, did not fester, did not rankle, did not bleed, did not drip; so may this wound by the virtue of that wound not hurt for long, nor be as whole and as clean as the wound that Longinus made in the side of our Lord Jesus Christ when he hung on the cross. In the name of the Father and of the Son and of the Holy Ghost. Amen.’

This text, known as the ‘Three Good Brothers Charm’, was very well known in the later Middle Ages.^41^ It incorporated material from another similar charm, known as the ‘Uncorrupted Wounds of Christ’ charm, and was believed to be an effective means of healing.^42^ The scribe drew attention to this item with the marginal marker ‘Charme’, and it is visually distinguished from the ordinary recipes that surround it by the frequent occurrence of crosses throughout the text (marked as  in the transcription), including one particularly elaborate marker that divides Christ’s name; these are prompts to show the reader that he must make the sign of the cross on his own body at these points. Thomas Roberts’s response to these texts was to treat them just like the other remedies. In the margin of f. 33r he has added the side heading ‘Charmes for the Tothe ache’, showing that he recognised the true nature of these texts. He indexed these charms along with the recipes in his list of contents, making no distinction between the two categories of material, except in his entry for the various prescriptions to staunch blood which reads: ‘for to staunche blode diuerse & a prayer’. The description ‘prayer’ for the charm reveals his positive regard for the text: to him this was an ordinary devotion, not superstitious or magical in any malevolent sense. The fact that these charm texts are not defaced in any way shows that members of the Roberts family believed them to be equally as effective as the other remedies, or at least held them in sufficient respect not to want to remove them. By contrast, the charms in Glasgow University Library MS Hunter 117 have been disturbed in various ways: the diagram to the ‘Plate of Lead’ charm on f. 36v has been obliterated, and Latin words in charm texts have been erased to frustrate incantation. In another late medieval vernacular collection of recipes in Cambridge University Library MS Dd.6.29 the charms have been systematically excised.^43^

## 5 Conclusions

It is not clear how the collection of medical recipes in BodL Rawlinson c. 299 came to be in the Roberts family library, but what is apparent is that Thomas Roberts had a high regard for its contents and made use of this book in the way that it was intended to function, namely, as a practical guide to maintaining good health. Collectively the additions and annotations within this small collection of medieval recipes reveal a sustained level of engagement with its written text by readers of a much more modern vintage than could have been expected by the volume’s original fifteenth-century compiler.^44^ The most significant additions to the manuscript were those contributed by Thomas Roberts, and, even though not all of the non-original recipes can be attributed to him, it is clear that in his eyes this old manuscript represented a store of currently relevant advice and practical application. Geoffrey Chaucer noted that ‘out of olde bokes, in good fey, / Cometh al this newe science that men lere’ (*out of old books, in good faith, comes new knowledge that men learn*).^45^ For Thomas Roberts and his family it was more a case of finding that the *old* science of old books was still relevant and applicable; despite their age, these medieval remedies were still needed, used, and valued in the early modern period. For these Tudor readers ‘old’ in this context was synonymous with reliable, tried and tested, and the old book was a source of authoritative wisdom, not outmoded knowledge.^46^ As modern scholars it is easy to overlook the value of old books, especially as we look back at the sixteenth century and view it as a period when printing had rendered manuscript production redundant, and when in historical terms we tend t o assess the state of knowledge by evidence that relates to dates of production. Many studies of early modern recipes, a booming field, have based their findings on printed materials.^47^ Yet print did not displace manuscript as rapidly as we tend to assume, and readers did not have the opportunities, nor the wherewithal (nor probably even the inclination), to replace all their manuscript books with printed copies.^48^ Medieval manuscript codices, especially those made up of parchment, were tough and enduring artefacts; in many cases they were long survivors in personal book collections.

## Footnotes

1. For examples of both see William H. Sherman, *John Dee: The Politics of Reading and Writing in the English Renaissance* (Amherst, MA: University of Massachusetts Press, 1995); and William H. Sherman, *Used Books: Marking Readers in Renaissance England* (Philadelphia, PA: University of Pennsylvania Press, 2008).

2. See, for example, Lisa Jardine and Antony Grafton, ‘ “Studied for Action”: How Gabriel Harvey Read His Livy’, *Past and Present*, 129 (1990), 30–78; Alison Wiggins, ‘What Did Renaissance Readers Write in Their Copies of Chaucer?’, *The Library*, 7th Series, 9 (2008), 3–36.

3. An observation made by Monica H. Green, ‘The possibilities of literacy and the limits of reading: women and the gendering of medical literacy’, in Monica H. Green (ed.), *Women’s Healthcare in the Medieval West* (Aldershot: Ashgate, 2000), VII.46. Certainly much scholarship in this field has focused on recipe books associated with elite women: see Elaine Leong, ‘Collecting Knowledge for the Family: Recipes, Gender and Practical Knowledge in the Early Modern English Household’, *Centaurus*, 55 (2013), 81–103, and the useful list of such studies given by Seth Stein LeJacq, ‘The Bounds of Domestic Healing: Medical Recipes, Storytelling and Surgery in Early Modern England’, *Social History of Medicine*, 26, 3 (2013), 454, note 11.

4. M. Teresa Tavormina, ‘The Twenty-Jordan Series: An Illustrated Middle English Uroscopy Text’, *American Notes and Queries,*18, 3 (2005), 40–64 has identified this as an incomplete copy of a Twenty-Jordan text (62), noting that it belongs to a recognised subgroup of this most common type of Middle English uroscopies. Jordans, or urine flasks, are drawn roughly at the start of each entry.

5. f. 4v, lines 11–12. See Figure 1. In all transcriptions from the manuscript, abbreviation marks have been silently expanded, and scribal punctuation and capitalisation have been modernised. Scribal spelling, including the Middle English letters ‘þ’ (‘thorn’) which represents ‘th’ and ‘ʒ’ (‘yogh’) which represents ‘gh’, has been retained. The medieval symbol that represents the measurement of an ounce has been rendered as ‘oz’. Modern English translations of the transcriptions are supplied in all instances except where confusion is extremely unlikely.

6. f. 4v, lines 13–18. In the first sentence ‘pastre’ is a scribal error and should read ‘plastre’. See Figure 1.

7. Green ointment was used on wounds to clean them and promote the growth of new flesh; its green colour derived either from herbs or from the addition of verdigris. Nerval was an ointment for the nerves or sinews.

8. A list of herbs effective against cancer in the mouth is given on f. 16r, after two prescriptions for the same problem.

9. See Melitta Weiss Adamson, *Medieval Dietetics: Food and Drink in the Regimen Sanitatis Literature, 800–1400* (Frankfurt am Main: Peter Lang, 1995); for an overview of dietary advice in the early modern period see Ken Albala, *Eating Right in the Renaissance* (Berkeley, CA: University of California Press, 2002).

10. There is general dietary advice to alleviate ‘þe rewme or þe pose’ (*cold or catarrh*) on f. 15r, and to prevent epilepsy on f. 21r.

11. On such efficacy statements see M.C. Jones, ‘Formula and Formulation: Efficacy Phrases in Medieval English Medical Manuscripts’, *Neuphilologische Mitteilungen*, 99 (1998), 199–209.

12. On texts of this nature see Irma Taavitsainen, ‘A zodiacal lunary for medical professionals’, in Lister M. Matheson (ed.), *Popular and Practical Science of Medieval England* (Ann Arbor, MI: Colleagues Press, 1994), 283–300.

13. ff. 30v–31v.

14. ff. 40v–41v, ‘Symple medecynes of gresys of rotys of sedys of gommys of metallys of oþer þinges of stonis …’ (*Simple medicines of grasses, roots, seeds, resins, metals, of other things, of stones* …); the first element listed is gold.

15. A fifteenth-century leechbook associated with a particular individual is London, British Library, MS Harley 1735, compiled by John Crophill of Wix, Essex. See Peter Murray Jones, ‘Crophill, John (d. in or after 1485)’, *Oxford Dictionary of National Biography*, http://www.oxforddnb.com/view/article/6780and Peter Murray Jones, ‘Witnesses to Medieval Medical Practice in the Harley Collection’, *Electronic British Library Journal* (2008), article 8. For an edition of the manuscript see L.G. Ayoub, ‘John Crophill’s Books: An Edition of British Library MS Harley 1735’ (unpublished DPhil thesis, Centre for Medieval Studies, University of Toronto, 1994).

16. For a more complete listing of both remedy books and leechbooks see George R. Keiser (ed.) *Works of Science and Information*, Vol. 10 of *A Manual of the Writings in Middle English 1050–1500*, ed. A.E. Hartung (New Haven, CT: Connecticut Academy of Arts and Sciences, 1998), 3653–8 and 3837–47.

17. Key editions in this field are: F. Heinrich (ed.), *Ein Mittelenglisches Medizinbuch* (Halle: Niemeyer, 1896); G. Henslow (ed.), *Medical Works of the Fourteenth Century Together with a List of Plants Recorded in Contemporary Writings, with their Identifications* (New York: Lennox Hill, 1899); H. Schöffler (ed.), *Beiträge zur Mittelenglischen Medizinliteratur*, Sächsische Forschungsinstitute in Leipzig, (Halle: Niemeyer, 1919); G. Müller (ed.), *Aus mittelenglischen Medizintexten: Die Prosarezepte des Stockholmer Miszellankodex X.90*, Kölner Anglistische Arbeiten, 10 (Leipzig: Bernhard Tauchnitz, 1929); W.R. Dawson (ed.), *A Leechbook or Collection of Medical Recipes of the Fifteenth Century* (London: Macmillan, 1934; repr. Whitefish MT: Kessinger, 2010); M.S. Ogden (ed.), *The ‘Liber de Diversis Medicinis’ in the Thornton Manuscript (MS Lincoln Cathedral A.5.2)*, Early English Text Society, Original Series 207 (London: Oxford University Press, 1938; rev. edn, 1969); T. Vallese (ed.), *Un ignoto ricettario medico inglese del XIV secolo trovato nella biblioteca nazionale di Napoli* (Naples: A.G.D.A., 1940); and most recently, Francisco Alonso Almeida (ed.), *A Middle English Medical Remedy Book*, Middle English Texts, 50 (Heidelberg: Winter, 2014).

18. A list of the ten collections which contain more than twenty-five manuscripts of scientific and medical texts is given by Linda E. Voigts, ‘Multitudes of Middle English medical manuscripts, or, the Englishing of science and medicine’, in M. Schleissner (ed.), *Manuscript Sources of Medieval Medicine: A Book of Essays* (New York: Garland, 1995), 183–95 (191).

19. Glasgow University Library, Hunter 185, recently edited by Almeida, *op. cit.* (note 17); the other manuscripts are Hunter 93 and Hunter 117.

20. Margaret Connolly, *The Index of Middle English Prose, Handlist XIX: Manuscripts in the University Library, Cambridge (Dd–Oo)* (Cambridge: D.S. Brewer, 2009), draws attention to several collections of medieval medical recipes in manuscripts in Cambridge University Library. The largest groups of recipes occur in MSS Dd.5.76 (two collections of 353 and 104 recipes), Dd.6.29 (226 recipes), and Ee.1.15 (two collections of 200 and 134 recipes); other leechbooks and remedy books in Cambridge University Library include MSS Dd.10.44, Ee.1.13, Kk.6.33, and Ll.1.18.

21. Glasgow University Library, MS Hunter 185 has the ownership inscription of Henry Swinburne (1560–1623), Bachelor of Civil Law, on a flyleaf, and several other seventeenth-century names, as well as the note ‘For Samuel Warnars sonne whoe hath a sore hedde’. There are some marginal drawings and numbers, and some words in the text have been underlined; see Almeida, *op. cit.* (note 17), 13.

22. The manuscript has been placed in Norfolk on the basis of its scribal dialect, see Angus McIntosh, Michael L. Samuels, and Michael Benskin, *A Linguistic Atlas of Late Mediaeval English*, 4 vols (Aberdeen: Aberdeen University Press, 1986), i.151. For an account of vernacular medical manuscripts derived from this region see M.C. Jones, ‘Vernacular Literacy in Late Medieval England: The Example of East Anglian Medical Manuscripts’ (unpublished PhD thesis, University of Glasgow, 2000).

23. For more details see Margaret Connolly, ‘Sixteenth-century readers reading fifteenth-century religious books: the Roberts family of Middlesex’, in Nicole R. Rice (ed.), *Middle English Religious Writing in Practice: Texts, Readers, and Transformations* (Turnhout: Brepols, 2013), 239–59.

24. Eight manuscripts may be connected with the Roberts family, *ibid*.

25. C.H. Talbot and E.A. Hammond, *The Medical Practitioners in Medieval England: A Biographical Register* (London: Wellcome Historical Medical Library, 1965) lists five individuals named Thomas Ward(e), of whom the closest to Thomas Roberts’s description seems to be Thomas Ward, surgeon of London who died in October 1521 leaving his estate to his son Christopher, although this Thomas Ward wished to be buried in the church of St Bride in Fleet Street; the church of St Andrew Undershaft was in Cornhill. The comments on Warde and on BodL Rawlinson c. 299 in R.S. Gottfried, *Doctors and Medicine in Medieval England 1340–1530* (Princeton, NJ: Princeton University Press, 1986), 62, are unreliable.

26. The *Middle English Dictionary* (*MED*) glosses walwort as a plant, usually Danewort or dwarf elder (sambucus ebulus), but cautions that the name could also be applied to other plants including chicory and hellebore.

27. At line 5 the scribe has supplied a word (‘anoynte’: *anoint*) that was accidentally omitted from the original text.

28. See Figure 2 where the number ‘xxiij’ has been added in the top margin. Late medieval manuscripts did not have original systems of page numbers, relying instead on quire signatures which were written at the bottom of the leaves in the first half of each gathering.

29. See the description of the creation of an early modern lawyer’s commonplace book in Sherman, *Used Books*, *op. cit.* (note 1), 127–48.

30. For an investigation of dirt in medieval manuscripts see Kathryn M. Rudy, ‘Dirty Books: Quantifying Patterns of Use in Medieval Manuscripts Using a Densitometer’, *Journal of Historians of Netherlandish Art*, 2 (2010), 1–26. Linne R. Mooney, ‘Manuscript evidence for the use of medieval English scientific and utilitarian texts’, in R.F. Green and L.R. Mooney, *Interstices: Studies in Middle English and Anglo-Latin Texts in Honour of A.G. Rigg* (Toronto: University of Toronto Press, 2004), 184–202, notes the evidence of blood stains in British Library MSS Sloane 100 and Additional 18216.

31. The inclusion of veterinary material in this type of manuscript is very common. For another example see Cambridge University Library, MS Ee.1.13, indexed in Connolly, *op cit.* (note 20), 86–94; details of many others may be found in the British Library online catalogue of the medical manuscripts in the Harley collection. See also the recent study by Briony Aitchison, ‘ “for to knowen here sicknesse and to do the lechecraft there fore”: Animal Ailments and their Treatment in Late Medieval England’ (unpublished PhD thesis, University of St Andrews, 2010), http://hdl.handle.net/10023/1024.

32. In the first line ‘the’ is a scribal error for ‘them’; see Figure 3.

33. See Mazhar Shah (ed. and trans.), *The General Principles of Avicenna’s Canon of Medicine* (Karachi: Naveed Clinic, 1966), 190–1; Michael C. Seymour (ed.), *On the Properties of Things: John Trevisa’s Translation of Bartholomaeus Anglicus De Proprietatibus Rerum*, 3 vols (Oxford: Clarendon, 1975–88), I.579. For a fuller account of late medieval beliefs concerning water see Carole Rawcliffe, *Urban Bodies: Communal Health in Late Medieval English Towns and Cities* (Woodbridge: Boydell, 2013), 188–97.

34. On humoral theory see Rawcliffe, *op. cit*. (note 33), 55–62.

35. See John L. Flood, ‘ “Safer on the battlefield than in the city”: England, the “sweating sickness”, and the continent’, *Renaissance Studies*, 17, 2 (2003), 147–76.

36. For a very similar Tudor recipe see Dawson, *op. cit.* (note 17), 339.

37. Theriac was a costly panacea imported from Italy for use against poison and the plague, see Christiane Nockels Fabbri, ‘Treating Medieval Plague: The Wonderful Virtues of Theriac’, *Early Science and Medicine*, 12, 3 (2007), 247–83.10.1163/157338207x20511518175463

38. This is followed by two lines in Latin. Another brief remedy against the plague, also in Latin but with an English heading ‘A preseruatif contra pestilent-’, is inscribed on f. 41v; this consists of only two lines of Latin that invoke the authority of John of Burgundy, the author of the well-known plague treatise *De epidemia* (or *De pestilentia*). See Lister M. Matheson, ‘Médecin sans Frontières? The European Dissemination of John of Burgundy’s Plague Treatise’, *American Notes and Queries*, xviii, 3 (2005), 17–27; for a modern English translation see Rosemary Horrox, *The Black Death* (Manchester: Manchester University Press, 1994), 184–93.

39. Such texts had a long history: see, for example, Tony Hunt, *Popular Medicine in Thirteenth-Century England* (Cambridge: D.S. Brewer, 1990). For a survey of the charms that occur in academic medical treatises see Lea T. Olsan, ‘Charms and Prayers in Medieval Medical Theory and Practice’, *Social History of Medicine*, 16, 3 (2003), 343–66.

40. See Keiser, *op. cit.* (note 16), 3671, no. 333; 3673, no. 351; and 3670–1, no. 328.

41. It occurs in French (‘Tres boni frates’) in the works of Gilbertus Anglicus (writing between 1200 and 1250) and John Gaddesden (*c.* 1280–1349), see Olsan, *op. cit*. (note 39), 364–5.

42. Keiser, *op. cit.* (note 16), 3672, nos 343 and 341.

43. See Margaret Connolly, ‘Practical Reading for Body and Soul in Some Later Medieval Manuscript Miscellanies’, *Journal of the Early Book Society*, 10 (2007), 151–74.

44. For an account of a seventeenth-century family recipe book of similar longevity see Elaine Leong, ‘Collecting Knowledge for the Family: Recipes, Gender and Practical Knowledge in the Early Modern Household’, *Centaurus*55, 2 (2013), 81–103.10.1111/1600-0498.12019PMC370912123926360

45. Geoffrey Chaucer, *The Parliament of Fowls*, lines 24–5, quoted from Larry D. Benson, *The Riverside Chaucer* (Oxford: Oxford University Press, 1985), 385.

46. The long-lasting nature of medical recipes, and the transmission of medical knowledge across centuries, has been explored, particularly in relation to women’s health, by scholars of antiquity, such as Helen King, and of the medieval and early modern periods, such as Monica H. Green and Mary E. Fissell, amongst others.

47. For example, Mary E. Fissell’s study of women’s reproduction, *Vernacular Bodies: The Politics of Reproduction in Early Modern England* (Oxford: Oxford University Press, 2004), focuses on the cheap print produced in sixteenth- and seventeenth-century England. The popularity of printed recipe books in the early modern period is summarised by William Eamon, *Science and the Secrets of Nature* (Princeton, NJ: Princeton University Press, 1994), 126–33.

48. For a recent assessment of the early years of print production in Britain, see Vincent Gillespie and Susan Powell (eds), *A Companion to the Early Printed Book in Britain 1476–1558* (Cambridge: D.S. Brewer, 2014).

